# Effectiveness of Hyperbaric Oxygenation Versus Normobaric Oxygenation Therapy in Carbon Monoxide Poisoning: A Systematic Review

**DOI:** 10.7759/cureus.5916

**Published:** 2019-10-15

**Authors:** Sebastian Casillas, Antonio Galindo, Luis A Camarillo-Reyes, Joseph Varon, Salim R Surani

**Affiliations:** 1 Research, Dorrington Medical Associates, Houston, USA; 2 Critical Care, United General Hospital, Houston, USA; 3 Internal Medicine, Texas A&M Health Science Center, Temple, USA

**Keywords:** carbon monoxide poisoning, hyperbaric oxygen therapy, normobaric oxygen therapy, haldane effect

## Abstract

Carbon monoxide (CO) is a gas product of combustion, considered highly poisonous. Prolonged CO exposure is responsible for more than half of fatal poisonings and is also one of the leading causes of poisoning in Western countries.

We aimed to compare the effectiveness of therapy with hyperbaric oxygen (HBO) versus normobaric oxygen (NBO) in the setting of carbon monoxide poisoning (COP). We independently searched the National Library of Medicine’s Medline (PubMed™), ScienceDirect™, and Scielo™ for any relevant studies published from 1989 to 2017, using the following keywords: hyperbaric therapy, hyperbaric oxygenation, normobaric therapy, carbon monoxide poisoning, carboxyhemoglobin, Haldane effect. We analyzed the studies that suggested the effectiveness of HBO or NBO. Also, we searched for studies related to COP; including history, epidemiology (risk factors, incidence, demographics), pathophysiology, clinical manifestations, diagnosis, and treatment.

Sixty-eight articles were found, sixteen of which dealt with either HBO or NBO or both. Twelve suggested HBO as the treatment of choice in COP; four studies indicated that NBO was an adequate treatment due to its cost-effectiveness and availability in the emergency department (ED).

HBO has been shown in several studies to be effective in moderate to high-risk COP situations, being the therapy of choice to avoid sequelae, especially neurologically. NBO can be considered as a reasonable alternative due to its cost-effectiveness. The availability and understanding of different therapeutic interventions are critical in the management of patients with COP in ED and the Critical Care unit.

## Introduction and background

Carbon monoxide (CO) is a colorless gas, with no smell or taste and is a product of combustion [1]. This gas is considered highly poisonous [2]. Common sources for CO include malfunctioning heating systems, the exhaust of internal combustion engines, incomplete combustion of fuels, and inhaled smoke [3,4]. High environmental concentrations of CO can be found in areas with poor ventilation and may cause signs and symptoms of intoxication [5,6]. Prolonged CO exposure is responsible for more than half of fatal poisonings, and is also one of the leading causes of poisoning in Western countries with several visits to the emergency department (ED) each year, just in the United States [5,7,8]. Hyperbaric oxygen (HBO) and normobaric oxygen (NBO) have been suggested as the key therapeutic options with several controversies presented between different studies regarding methods, the number of patients, absolute atmospheres (ATA) given during HBO therapy and overall clinical benefit [9]. One of the most widely quoted papers is the study by Raphael and colleagues who performed a randomized trial, assessing the controversial and debated topic regarding therapeutic advantages and indications in the treatment of CO poisoning with HBO, compared with the classical paramount management with NBO [10]. Six hundred and twenty-nine adult patients were included in this study; inclusion criteria consisted of CO poisoning (COP) within 12 hours prior to admission. The studied population was divided into four groups based on the level of consciousness for which HBO and NBO were administered alone or in combination. The study concluded a reduction in neurological sequelae in those patients that sustained a loss of consciousness and attributed this effect to the wider use of HBO [10].

The clinical manifestations of CO poisoning are highly unspecific; these include headache, myalgia, dizziness (in severe exposures, confusion), loss of consciousness, and death [4,7]. CO binds to hemoglobin 200-230 times more effectively than oxygen. This causes a leftward shift in the oxygen-hemoglobin dissociation curve, decreasing oxygen delivery to the tissues; the resulting tissue hypoxia has been proposed as the main mechanism of CO toxicity. CO additionally affects the cerebral perfusion through altered vasoactivity and neuronal responses independent of hypoxic stress [1]. The resulting generation of carboxyhemoglobin produces a left shift dissociation on the oxygen-hemoglobin curve. This was, first described by Claude Bernard in 1865 [8].

Prolonged CO exposure increases cytosolic heme levels, which leads to oxidative stress and binds to platelet heme protein and cytochrome c oxidase, which interrupts cellular respiration, causing increased reactive oxygen species generation leading to neural necrosis and apoptosis [7]. Given its highly unspecific clinical presentation, CO poisoning remains one of the most challenging medical conditions to diagnose, the key element to this puzzle remains on the high level of suspicion that the physician must have based on a detailed history, including symptom duration and correlation with environmental CO exposure [11,12]. Current methods to properly diagnose CO poisoning reside in the measurement of carboxyhemoglobin (COHb); it is a useful diagnostic method but is considered a weak indicator of the severity of poisoning, a consequence of the delay of the blood assay and timing of poisoning occurrence [9,13]. The purpose of this article is to systematically review these two therapeutic interventions and compare their effectiveness.

History

The Greek and Roman empires made the first documented registry of CO application; both civilizations were familiar with this gas toxicity and found ways to apply its properties in the punishment of criminals. It was in 1880 when William Cruikshank described the main chemical properties of CO. In the middle of the 19th century, French physiologist Claude Bernard recognized that CO caused hypoxia by direct interaction with hemoglobin, and at the end of the century, Haldane studied the relationship between the high partial pressure of oxygen and its therapeutic effect on hemoglobin and CO [14].

Epidemiology

Prolonged CO exposure represents one of the most common causes of unintentional intoxication worldwide [15,16]. It is mainly seen in a residential setting in the US and western countries. The high occurrence is confirmed by the Centers for Disease Control and Prevention (CDC) with an incidence of 23.2/million population per year and nearly 15,000 emergency department visits and 500 deaths each year in the United States alone [17]. Most of the CO exposures occur at home, and mostly involve females, children aged ≤17 years, and adults aged 18-44 years. On analysis of data regarding CO exposures, males represented an alarming 74% of unintentional non-fire related deaths and significant long-term morbidity, with an estimated 50% of neurologic sequelae in the non-fatal cases [8].

Pathophysiology

CO intoxication impairs oxygen delivery to tissues, resulting in cellular ischemia [18,19]. It is explained by the tetrameric structure of the hemoglobin molecule and the affinity among the heme groups when the CO is bound at one of its four sites, which causes a left shift of the oxygen-hemoglobin dissociation curve and a hyperbola-like shape [15]. The latter results in a hemoglobin molecule that has a decreased capacity to release oxygen to the tissues, including the respiratory center, leading to acidosis. CO causes mitochondrial dysfunction due to binding to the cytochrome c oxidase, leading to worsening of hypoxia, and increasing the production of reactive oxygen species by alteration of the electron transport chain [19,20]. In severe CO exposures (3000 ppm CO), the cAMP pathway has a deleterious role in ischemia-induced brain-damage [21].

Clinical presentation

The symptoms of CO poisoning are usually nonspecific and can imitate a wide variety of disorders [7,14]. Oxygen-dependent organs and those with the lowest metabolic stores show the initial signs of injury, such as the brain and heart [5]. The severity of CO poisoning depends on several aspects: exposure to this gas, being the most important, the concentration of molecules of CO in the air, and the observed level of COHb. In acute poisonings, a concentration below 1000 parts per million (ppm) and a COHb of 10% typically results in headache; concentrations above 1000 ppm and COHb of 20% results in dizziness, irritability, dyspnea, nausea, confusion, and syncope [22]. In patients with exposure from 3000 ppm and more than 50% of COHb, seizures, coma, and death may occur after prolonged exposure [23]. Myocardial injury and fibrosis have been reported after chronic exposure to moderate CO concentration; arrhythmias and sudden death (especially among patients with preexisting cardiac conditions) have been associated with acute environmental exposure (up to 500 ppm) [16]. It has been suggested that delayed CO encephalopathy can cause cognitive impairment and memory loss, and neuropsychologic symptoms, days to weeks after the exposure [24-26]. The classic cherry red hue that is commonly associated with CO poisoning patients is an uncommon post-mortem sign and is rarely appreciated in the clinical assessment [8,15].

Diagnosis

The diagnosis of COP rests in the high level of suspicion based on a detailed history of events, a key component of the initial assessment. Several diagnostic methods have been developed with the purpose of a rapid diagnosis, currently, the measurement of COHb by blood gas analysis represents the standard approach to a patient with a possible gas intoxication, several disadvantages of this method resulted from delayed blood assays and unavailability of first respondents in non-hospital scenarios have made clinicians look for other options for a timely diagnosis of the poisoning [8,17,18,27]. In recent studies, the efficacy of CO oximeters and exhaled CO analyzers during the initial assessment have been remarkable in diagnosing the most severe poisoning cases in very short periods of time [13,17,27].

## Review

Materials and methods

Data Sources

This study protocol was prepared following PRISMA guidelines. We sought to identify all relevant manuscripts that compared COP treatment with HBO and NBO. Our objective was to demonstrate the outcome with different therapeutic interventions. We searched electronic medical databases (PubMed™, ScienceDirect™, and Scielo™) and excluded studies that did not provide the outcome data. The authors independently searched relevant studies published from December 1989 to March 2017. All types of articles, including comprehensive reviews, prospective observational studies, randomized controlled trials (RCTs), multi-group comparison, editorials, retrospective studies, and case reports, were consulted for analysis of relevant content.

Data Search

The following keywords were utilized: hyperbaric oxygenation therapy, normobaric oxygenation therapy, carbon monoxide poisoning, carboxyhemoglobin, and Haldane effect. The articles were selected if they reported outcome data as well as complications, sequelae and/or mortality. The references quoted in the selected articles were also reviewed independently and added for further analysis and comparison between HBO and NBO outcomes. Issues regarding article eligibility for data collection were resolved by discussions with another member of the team.

Results

**Figure 1:**
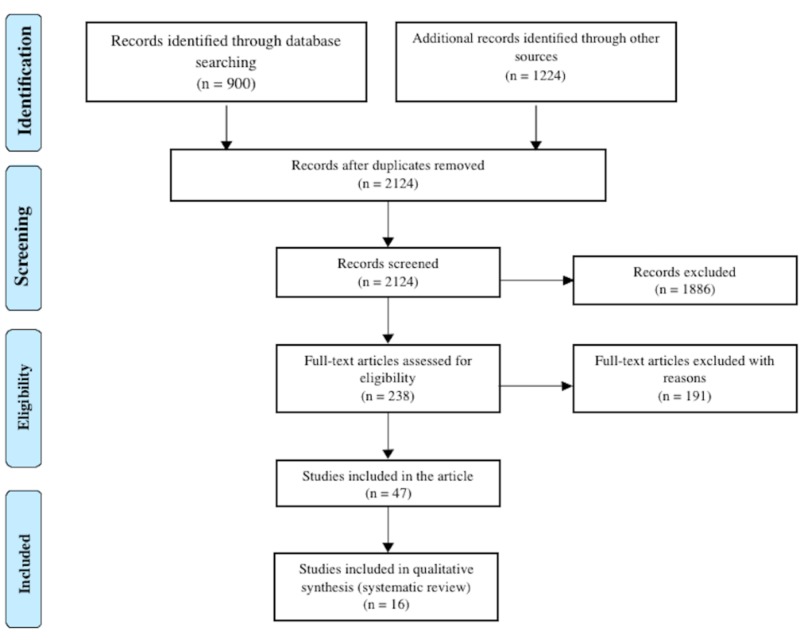
Flow diagram for study selection according to PRISMA 2009 guidelines

A total of 2124 articles were found from database searching and other sources and later scrutinized for any data related to carbon monoxide poisoning treatment with hyperbaric therapy and normobaric therapy (Figure 1). Only 16 clinical studies dealt specifically with outcomes after HBO/NBO therapy for COP. Twelve of them showed HBO as the therapeutic intervention of choice in COP; four studies showed that NBO was a reasonable method due to its cost-effectiveness and availability in the ED. 

Discussion

The highly unspecific clinical presentation of carbon monoxide poisoning (COP) entails a delayed diagnosis and treatment that represents an important impact on the probability of short and long-term neurological sequelae that are directly related to an early cognitive decline. In the last two decades, several studies have been focused on expedited diagnosis which in turn leads to prompt treatments and possibly better outcomes. The main controversy has been whether to use HBO or NBO. Historically, Henshaw in 1662, first applied compressed air, trying to prove the HBO theory but the experiment barely reached the pressure of 1.3 atm due to the inefficiency of his equipment [28]. This therapy elevates arterial and tissue oxygen tension, therefore, it enhances the elimination of carbon monoxide, meanwhile, increasing the adenosine triphosphate (ATP) production, reducing the oxidative stress and inflammation [7,8,29].

The half-life of the COHb is three to four hours when breathing room air, which is reduced to 30 to 90 min in the presence of 100% oxygen (normobaric oxygenation) and to 15 to 23 minutes with HBO at 2.5 atm [30]. The use of HBO has been demonstrated in previous studies that it significantly reduces the rate of complications, neurological sequelae, and mortality. In 1999, Camporesi demonstrated a decline in mortality from 30% to 13.5%, in a group of 213 patients; none of the patients treated with HBO suffered from sequelae [31]. Garrabou et al. proved the effectiveness in recovering mitochondrial complex IV (mtCIV) function depends on the oxygen therapy that was administered [29]. Having said that, since HBO was more effective than normobaric oxygenation (NBO) in moderate and severe acute COP, one session of HBO was effective enough to restore mitochondrial activity reducing the rate of possible complications [20,29,32].

Researches have concluded that the molecular effect of HBO results in the prevention of lipid peroxidation in the central nervous system and preservation of ATP levels in tissues exposed to carbon monoxide, therefore reducing the damage [33-35]. Evidence suggests that HBO can reduce neurological/cognitive sequelae of COP if administered within 24 hours of an acute episode of CO intoxication [36-38]. Vomero et al. analyzed three clinical cases of CO poisoning, in which the three patients were found unconscious; they mentioned that HBO must be applied under certain criteria such as loss of consciousness, neurological disturbance or coma [39]. This being the case, the patient must be transferred safely and hemodynamically stable [40,41]. A prospective trial demonstrated that the incidence of cognitive sequelae was lower in patients who underwent three HBO sessions within 24 hours after COP compared with patients treated with NBO [8,33]. The Undersea and Hyperbaric Medical Society, recommends HBO therapy for patients with serious CO poisoning as manifested by unconsciousness, abnormal neurologic signs, cardiovascular dysfunction such as myocardial infarction and acute coronary syndrome, severe acidosis or patients who are older than 36 years of age that were exposed for more than 24 hours, displaying a carboxyhemoglobin level of 25% or more. Thom and colleagues performed a randomized control trial on 60 patients (divided into two groups) with mild-moderate COP presented during the first six hours post-exposure. Neurologic sequelae were seen in 23% of patients in the control group and none in the HBO group [15,42]. Ducasse et al. performed a similar study in 26 non-comatose patients with acute COP divided into two groups and at 12h none of the patients in the HBO group (0/13) had abnormal clinical findings, compared to the NBO group with 5/13 [43]. In 2017, Huang and colleagues showed that patients with CO poisoning who received HBO therapy had a lower mortality rate than those who did not, especially patients who were younger than 20 years and those with acute respiratory failure. In this study, patients under 20 years of age with acute respiratory failure had even more reduction in the mortality risk after HBO [44]. Also, a large-scale randomized controlled trial in Taiwan studied 25,000 patients with COP over a 13-year time frame; 7000 underwent HBO and they found improvement in mortality rate against those patients who underwent standard therapy (NBO) [45]. Several studies involving patients with different clinical settings suggested certain benefits in overall outcomes with the application of HBO at 2 or 3 ATA, and one clinical trial reporting superior outcomes with 3 ATA [9].

In 2015 the prevalence of CO exposure in pregnant women increased to 8.5% and this was associated with a mortality rate between 19% and 24% and a fetal mortality rate between 36% and 67% [5]. Friedman performed a review of literature among pregnant patients and concluded that HBO therapy is appropriate since these patients should generally require five times the length of the treatment in order to avoid sequelae in both, the mother and the fetus [5].

NBO hastens the elimination of carbon monoxide and is safe, easily available and inexpensive. If used as a single-agent treatment, it should be provided until the carboxyhemoglobin level is less than 5%. Evidence suggests that NBO may be less effective than HBO for preventing cognitive sequelae. A prospective study showed that 34% of NBO-treated patients reported symptoms such as headaches or memory problems at four weeks and 46% of the patients had neuropsychological sequelae at six weeks [7]. Another study showed that NBO was the appropriate therapy, even though the patients developed neurological symptoms at about six weeks after treatment; it also suggested that the treatment with HBO has been a controversial issue, and precise guidelines must be established in order to be applied [46]. Sen et al. established that the treatment of CO intoxication first requires a high-flow of 100% NBO until COHb is normalized [3]. Another study performed by Scheinkestel refuted the use of HBO in CO poisoning; they provided a daily 60 minute treatment at 2.8 ATA on three consecutive days for the HBO group in conjunction with prolonged high flow oxygen therapy between treatments (this group received oxygen therapy equated to approximately 35.7 COHb-dissociation half-lives, while the NBO group received the equivalent of 28.5 COHb-dissociation half-lives), while the NBO group received at least three consecutive days continuous oxygen by non-occlusive facemask at 14 L/min [47]. The authors attributed the worse outcome in the HBO group to the higher doses of oxygen that may add no further benefit and possibly causing adverse effects. This study had a low follow up rate (only 46% of patients attended the one month follow up). We excluded this paper from further analysis since no clinical evaluation was assessed to rule out sequelae or for any clinical outcome in both groups [47].

The management of CO poisoning eliminates carbon monoxide from the body and increases the oxygen content of the blood by the means of administration of high flow and concentration of oxygen, ventilatory support, and monitoring for abnormal cardiac rhythms [7,15]. Most of the studies in this review showed that HBO had greater therapeutic efficacy over NBO (Table 1). HBO was appropriate for life-threatening CO poisoning with COHb levels greater than 15%, and in patients with a history of loss of consciousness, neurological symptoms, pregnancy or cardiac compromise [5]. The disadvantages of HBO therapy included barotrauma, hypoxic seizures due to high intake of oxygen in a short period of time and those risks associated with the transport of patients to treatment centers [8,34,37].

**Table 1:** HBO and NBO studies comparison Abbreviations: HBO, hyperbaric oxygenation; NBO normobaric oxygenation; ROL, review of literature; PS, prospective study; MGC, multi-group comparison; RCT, randomized control trial; CR, case report; RCS, retrospective cohort study; CO, carbon monoxide; COHb, carboxyhemoglobin; ACOP, acute carbon monoxide poisoning

Studies supporting the effectiveness of HBO			Studies supporting the effectiveness of NBO		
Article	Type of study	Comments	Article	Type of study	Comments
Koren et al. 1991 [6]	PS	Reduce the rate of spontaneous abortion in pregnancy with CO poisoning. In two Cases were COHb was 39% and 21%, HBO was applied and normal outcome was seen in the 1st year of life.	Sen et al. 2010 [3]	CR	NBO should be administered until COHb is normalized, since HBO usage is controversial.
Jang et al. 2017 [20]	PS	Improved mitochondrial dysfunction.	Weaver L. 2009 [7]	CR/ROL	High-flow 100% oxygen is safe, available and inexpensive compared to HBO.
Garrabou et al. 2011 [29]	PS	HBO was more effective than NBO in moderate ACOP, and promotes up to 32% of mitochondrial recovery.	Bor-Kucukatay et al. 2010 [34]	MGC	NBO caused a decrement in red blood cells aggregation, HBO caused increment of free radical production.
Camporesi 1999 [31]	ROL	HBO started in the first six hours, decreased mortality from 30% to 13.5%.	Bartolome et al. 2010 [46]	ROL	NBO is the treatment for election with the proper follow up.
Jurič et al. 2015 [32]	MGC	HBO reduced toxic effects of CO in astrocytes while NBO showed no beneficial effect.			
Weaver et al. 2002 [33]	RCT	Reduced the cognitive sequelae by 46%.			
Perez et al. 2017 [35]	CR/ROL	The only effective treatment to avoid delayed neuropathy, since decreases COHb to 23 minutes (against 74 minutes on NBO).			
Santiago I. 2003 [37]	ROL	Decreased lipid peroxidation in the brain and reduced neurological sequelae.			
Lueken et al. 2006 [38]	CR	Improvement in cognitive and neuropsychiatry symptoms after one session.			
Thom et al. 1995 [42]	RCT	In 60 patients with mild-moderate COP presented within six hours. Neurologic sequelae was seen in 23% of control group and none in the HBO group.			
Ducasse et al. 1995 [43]	RCT	26 non-comatose patients with acute COP were divided in two groups. At 12 h no patient in the HBO group (0/13) had abnormal clinical findings, compared to the NBO group with 5/13.			
Huang et al. 2017 [44]	RCS	More than one session of HBO reduced mortality in patients <20 years old.			

## Conclusions

Our systematic analysis showed that HBO is effective in moderate to high-risk situations and should be the therapy of choice to avoid sequelae, especially neurologically. This type of treatment has been useful in the last decades due to its powerful effect to counteract the toxicity of carbon monoxide as it decreases the half-life of carboxyhemoglobin in the blood-stream considerably, reducing it to less than a quarter of its normal physiological half-life. It is widely known that not every medical center has hyperbaric chambers due to its cost and expertise, therefore, NBO can be considered as a viable therapeutic option, presenting acceptable outcomes in comparison with HBO.
